# The impact of clinical placements on the emotional intelligence of occupational therapy, physiotherapy, speech pathology, and business students: a longitudinal study

**DOI:** 10.1186/s12909-019-1520-3

**Published:** 2019-03-27

**Authors:** Nigel Gribble, Richard K. Ladyshewsky, Richard Parsons

**Affiliations:** 1 School of Occupational Therapy, Social Work, and Speech Pathology, Curtin University, GPO Box U1985, Perth, 6845 Australia; 2 School of Management, Curtin University, Perth, Australia

**Keywords:** Clinical placements, Emotional intelligence, Therapy students, Supervision

## Abstract

**Background:**

Emotional intelligence (EI) is a critical skill for healthcare practitioners. Minimal longitudinal research has tracked the changes in EI of therapy students over their final full-time clinical placements.

**Methods:**

The Emotional Quotient Inventory (EQ-i^2.0^) measured the EI of 283 therapy students and 93 business students (control group who do no clinical placements) at three time points over a 16-month period, the same period that the therapy students participated in clinical placements.

**Results:**

Analysis of the therapy students showed significant increases over the 16 months of the study in Total EI score, as well as nine other EI skills. However, large percentages of students reported declining scores in emotional-expression, assertiveness, self-expression, and stress tolerance, with some students reporting low EI scores before commencing full-time extended clinical placements.

**Conclusions:**

The study contributes to new knowledge about the changing EI skills of therapy students as they complete their full-time, extended placements. Emotional intelligence in student therapists should be actively fostered during coursework, clinical placements and when first entering the workforce. University educators are encouraged to include EI content through the therapy curricula. Employers are encouraged to provide peer coaching, mentoring and workshops focused on EI skills to recent graduates.

## Background

Emotional intelligence (EI) is of fundamental importance to many aspects of human functioning with MacCann, Joseph, Newman, and Roberts [1] arguing that EI should be included as a second-stratum factor of intelligence and considered as important as visual processing and fluid intelligence. Emotional intelligence is the “…abstract, invisible processes that people appear to use in their relationships with themselves, and as part of their relating effectively, meaningfully or helpfully with others” ([2], p. iv). Morrison [3] proposes that EI skills are required when healthcare practitioners develop rapport with patients and families, make decisions during assessment and treatment, collaborate with the healthcare team, and cope with workplace stress. Evidence has demonstrated that healthcare teams with higher EI skills have enhanced therapeutic outcomes with patients [4]. Emotional intelligence has been shown to have a significant effect on patient-centred care [5], patient satisfaction [6], job satisfaction [7], staff retention [8], and team skills [9]. Medical students with higher EI scores performed better than students with lower EI in their final professional examination with the authors reporting that EI development may enhance medical students’ academic performance [10]. Two studies [11, 12] reported that Total EI scores of occupational therapy students positively correlated with their performance during clinical placements. Many healthcare and business graduates aspire to management and leadership roles with research reporting that leaders with higher EI skills are often more effectual [13].

Emotional intelligence tends to increase as each individual matures emotionally. Many studies have tracked the emotional-social development through childhood. Zeidner et al. [13] proposed a range of biological, social and environmental factors including personal experiences, peers, affective interactions, teachers and school and the media, that work synergistically in the development of EI skills. Fewer studies have followed the trajectory of EI development in adults. Bar-On’s [14] study showed that EI increases from the late teenage years, through adulthood but plateaus in the late forties. Multi-Health Systems [2] also showed EI increased from the teenage years and well into the sixties. Research has shown that EI can be increased through participation in training programs [15–17] as well as via workplace learning [18]. Workplace learning occurs when a worker or student performs the actual job in an authentic workplace. Mechanisms that lead to workplace learning include role-modelling from expert colleagues, mentoring, coaching, and team collaboration. Workplace learning has been shown to develop a range of complex skills deemed necessary for the twenty-first-century workplace, especially problem-solving, creativity, and teamwork [18]. As a result of the finding that EI can be enhanced via training, some authors [19, 20] have advocated for university allied health and medical programs to embed EI throughout the curriculum, so students enter the workforce equipped with improved EI abilities. The reality appears to be that university healthcare programs include minimal EI content in their curricula [19, 20]. Thus, the most fertile ground where healthcare students learn EI skills may be during clinical placements.

Various studies have demonstrated that the EI skills of healthcare students’ can improve during their university course. Foster et al. [19] reported that nursing students’ (*n* = 111) Total EI increased significantly over their three-year program. Benson et al.’s [21] longitudinal study examined changes in EI of 52 nursing students over their entire university program. Although this study reported that Total EI scores did not change significantly, specific EI skills did change significantly. Lewis [22] followed 87 physiotherapy students over 3 years and found that their EI scores did not change significantly; however, those students who failed their licensure exam tended to have lower EI scores. Similarly, Larin et al. [23] found no significant change in EI scores amongst 73 nursing and 60 physiotherapy students from the commencement of their program to after their first clinical placement. While the literature presents some conflicting results in the changes of EI scores of healthcare students, none of the studies used a control group of relatively homogenous university students against which to compare any changes in EI over time. Thus, the studies are unable to conclude that the changes (or lack of change) in EI scores were a result of university studies or because of personal life events external to the university studies. Nor did these studies focus their measurements over the final period of the university program where the majority of clinical placements typically occur. As such, we decided to longitudinally track EI scores of occupational therapy, physiotherapy, and speech pathology students before, during and after they completed their final clinical placements, and compare results for these students with those for business students, who do no clinical or work placements. The research hypothesis was: the Total EI, as well as the Composite and Subscale EI scores of therapy students (who completed clinical placements), will improve significantly more than the business students (who do no clinical or work placements), with business students’ EI scores expected to show no significant change.

## Methods

This paper reports the findings of the quantitative phase of a larger study which used a longitudinal, retrospective mixed methods design as proposed by Plano Clark et al. [24]. An analysis of the therapy students’ baseline scores before the commencement of clinical placements [25], their changes in the EI skills from T1 to T2 [26], and the qualitative findings [27] have been previously published.

### Participants

Participants were recruited from a convenience sample of undergraduate students enrolled at four Australian universities.

To be eligible to participate in the study, students needed to be enrolled in the third-year of their four-year undergraduate university occupational therapy, physiotherapy, or speech pathology program at one of the four selected universities. The three therapy professions were selected as they work with similar patients across a range of healthcare settings. Undergraduate business students were selected as the control group as they generally undertake minimal or no placements in healthcare settings as an enforced component of their program, although work integrated learning in business programs is becoming more popular [28]. To be eligible, business students needed to be enrolled in the second-year of their three-year commerce, economics, or human resource management program.

### Data collection

Data were collected at three time points. The first set of data collection (T1) occurred before the therapy students commenced their full-time, extended clinical placements. The second set of data collection (T2) was completed the following year after the therapy students had completed one or more clinical placements (7 to 8 months after T1). The final set of data (T3) was collected after the therapy students had completed all mandatory placements (7 to 8 months after T2). The T1, T2 and T3 demographic data were collected via an online survey tool and students were then directed to the Multi-Health Systems website to complete the EI questionnaire. Therapy and business students completed the online questionnaires at the same times. The online questionnaires were available for a period of 5 weeks. At T2 and T3, students received four emails that requested their continued participation in the study.

**Fig. 1:**
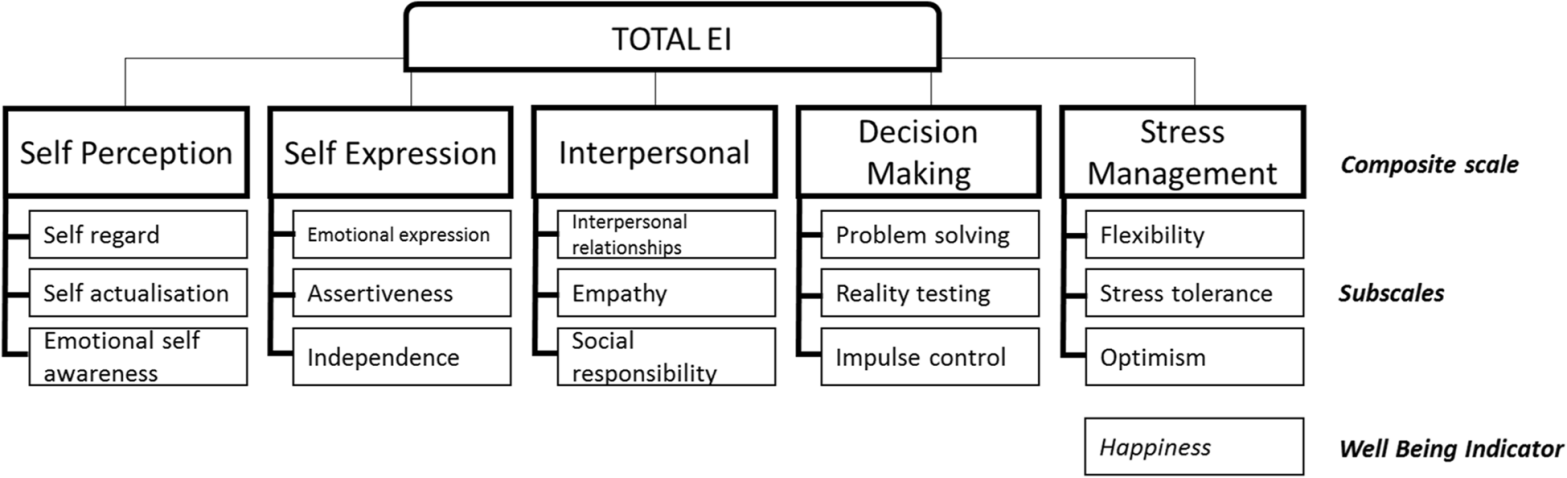
Model of Emotional Social Intelligence showing the Composite, Subscales and Well-Being Indicator (adapted from Multi-Health Systems, 2011)

When selecting a framework on which to base this study, two theoretical perspectives of EI were considered; ability-based and mixed models, each of which has their own measurement tools [29]. Ability models propose that EI is an individual’s ability to perceive emotions, generate emotions to assist thought, understand and interpret one’s and others emotions, and to be able to regulate emotions [30]. Mixed models purport that, compared to the ability models, EI draws on a broader range of skills including personality and motivational traits that enable a person to use emotions effectively in day-to-day life [14, 31]. For this study, a mixed model, Bar-on/Multi-Health System’s Model of Emotion Intelligence [2, 14] was preferred as the researchers believe this model underpins many of the abilities and skills required by therapists and in students working in healthcare settings. The Model of Emotional Intelligence includes skills such as self-regard, assertiveness, flexibility, and stress tolerance; skills that therapists require to work effectively with patients in vulnerable situations. Happiness is included in the model as a Well-Being Indicator because research has reported that happiness is higher in people with higher EI. The subsequent results of our study do not include the happiness scores as happiness is an outcome of higher EI, not a contributing factor [2] Fig. 1.

To measure EI, Bar-On [14] created the Emotional Quotient Inventory (EQ-i). In 2011, Multi-Health Systems revised the measurement tool, now titled Emotional Quotient Inventory 2.0 (EQ-i^2.0^). The EQ-i^2.0^ is a 133 item self-report tool. Thus the instrument does not purport to measure the student’s actual EI ability but their perceptions of their emotional intelligence. The EQ-i^2.0^ asks participants questions related to the five Composites and 15 Subscales of the Model of Emotional Intelligence. Questions include: “I’m aware of how others feel”, “I can’t think clearly when I’m under stress”, and “It’s hard for me to share my feelings with others”. Each question is answered on a five-point scale from ‘Never/Rarely’ through to ‘Always/Almost Always’. The online test takes 20 min or more to complete. The EQ-i^2.0^ calculates 22 standard scores for each participant: a Total EI score, five Composite scores, 15 Subscale scores, and the Well-Being Indicator score. The EQ-i^2.0^ scoring manual [2] states that the standard scores are attained by converting raw answers for all EI Subscale and Composite scores to scores whose population mean is 100 with a standard deviation of 15.

During analysis of EI, scores above 110 are considered high, scores between 90 and 110 are considered normal, and scores below 90 are considered low. These demarcations were arrived at during the normative phase. The highest score possible on each Subscale and Composite is 135 and the lowest is 55. Test-retest reliability for Total EI has been reported to be high (r = 0.92) for subjects with 2 to 4 weeks between tests and lower (r = 0.81) when tested 8 weeks apart [2].

In order to track changes in EI, the EQ-i^2.0^ manual recommends that retesting occur at least 3 months apart. Australian normative data for the EQ-i^2.0^ was used in this study [32].

### Data analysis

Statistical analyses were performed with the SAS version 9.2 software [33] and a *p*-value< 0.05 was taken to indicate a statistically significant association in all tests. Comparisons of EI scores (Total, Subscales and Composites) between the three therapy groups at baseline (T1) was conducted with either ANOVA or Kruskal-Wallis tests, depending on the Normality of the baseline distributions (tested with the Shapiro-Wilk statistic). The changes in all subscale and composite scores from T1 to T3 were calculated for each participant. As the changes in scores were found to be close to Normally distributed, a t-test was used to identify whether there was a significant change in mean total EI score from T1 to T3 for each student group (testing whether the change for each group was statistically different from zero). Instead of performing a number of separate t-tests on the Subscale and Composite scores, one analysis was performed on the five Composites and another on the 15 Subscales. These were carried out by treating the measurement on each Subscale and Composite as a repeated measurement for each participant, with the type of measure (particular Subscale and Composite) as an independent fixed factor. Because of the repeated measurements on each participant, the participant identifier was treated as a random effect in the model. The results from this model were considered more stable than conducting many separate t-tests, as the estimated standard deviation against which all the tests are performed would be obtained from consideration of all the Subscale and Composite scores together. With the participant identifier named as the random effect, any correlation between scores obtained from the same participant could be taken in to account. By including an interaction between the score type and the student type, *p*-values were obtained to identify whether there had been any significant change from T1 to T3 (interaction term significantly different from zero) for each particular student type and score type (Subscale and Composite) combination. A similar model was used to compare changes in the therapy students as a single group against the changes observed in the business students (pairwise differences between selected interaction terms in the model).

The scores for each participant were classified as increased, no change, or decreased from T1 to T3, depending on whether the change had exceeded a five-point threshold or not. This margin of five-points was selected because the study by Larin et al.’s [34] was able to detect an effect size of 0.36 for the total EI score, corresponding to a change of approximately five-points.

## Results

At T1, 650 third-year therapy students and 750 s-year business students were enrolled in the eligible courses at the four universities. All were invited via email and face-to-face recruitment sessions to take part in the study. A total of 283 therapy and 93 business students completed all parts of the online questionnaires and were included in the data analysis at T1. By the third and final questionnaires, the retention rate was 50% (*n* = 142) for therapy and 26% (*n* = 24) for business students. The higher than expected drop-out rate was due to the collection of T3 data being after students completed their university programmes and thus they were less likely to view and respond to emails requesting their ongoing participation in the research study. Consequently, with the business students, the study may have lacked the power to detect changes from T1 to T3 which increases the chance of a Type II error. The EQ-i^2.0^ calculates an Inconsistency Index, Positive Impression and Negative Impression score, with 11 participants exceeding the parameters at one or more of the three data collection points and excluded from the data analysis.

Table 1 details the clinical placements completed by the students over the 16-month period. The business students completed no workplace placements. Therapy students completed a mean of 124 days of placements in a range of settings, including hospital, private practices, schools, and residential aged care facilities, in rural, and international locations. The majority (94%) of placements for therapy students were 5 days per week.

**Table 1:** Participants demographics and clinical placements at T1 and T3

	All therapy students		Occupational Therapy		Physiotherapy		Speech Pathology		Business	
	T1	T3	T1	T3	T1	T3	T1	T3	T1	T3
Participants	283	142	139	52	91	53	53	37	93	24
Females and males @T1	85%/15%		89%/11%		72%/28%		96%/4%		76%/24%	
Age @ T1	21.4 years SD = 3.4		21 years SD = 2.7		21.9 years SD = 3.7		21.4 years SD = 3.5		21.4 years SD = 4.7	
Number of clinical placements	4.02 SD = 1.4		3.24 SD = 0.9		4.5 SD = 1.5		3.9 SD = 1.2		0	
Clinical placement days	124 SD = 33		117 SD = 28		125 SD = 37		124 SD = 37		0	

Table 2 presents each student cohorts’ T1 scores and the mean change from T1 to T3 in total EI scores as well as Composite and Subscale scores with bolded *p*-values indicating that a significant positive change occurred from T1 to T3. At T1, before therapy students commenced their first full-time, extended placements, the occupational therapy students’ mean independence score was low (< 90), while speech pathology students reported mean scores that are considered low for independence, problem-solving and stress tolerance.

**Table 2:** EI scores for all participants at T1, change in EI scores between T1 and T3

	Occupational Therapy			Physiotherapy			Speech Pathology			Business		
	T1 score (SD)	Change from T1–3	^X^ *p*-value	T1 score	Change from T1–3	^X^ *p*-value	T1 score	Change from T1–3	^X^ p-value	T1 score	Change from T1–3	^X^ p-value
Total EI Score	99 (12)	3.2	0.034	99 (15)	2.5	0.121	97 (12)	2.2	0.286	96 (14)	−1.7	0.445
SELF PERCEPTION	101 (3)	3.8	**0.016**	100 (14)	2.6	0.136	99 (12)	1.5	0.483	98 (14)	−1.3	0.579
Self-regard	97 (14)	2.5	0.146	97 (16)	2.4	0.209	93 (14)	1.4	0.542	97 (15)	−1.4	0.593
Self -actualization	104 (14)	3.5	**0.038**	103 (15)	4.5	**0.017**	102 (12)	1.9	0.420	97 (15)	−0.2	0.937
Emotional self- awareness	103 (13)	1.6	**0.032**	101 (14)	−1.3	0.500	105 (12)	0.5	0.830	103 (15)	−1.6	0.543
SELF EXPRESSION	94 (14)	2.4	0.128	95 (15)	1.8	0.286	93 (14)	1.6	0.447	96 (14)	−2.4	0.316
Emotional expression	102 (15)	1.6	0.353	99 (16)	−1.3	0.498	102 (14)	3.12	0.179	101 (15)	−1.4	0.596
Assertiveness	95 (15)	−1.5	0.367	97 (15)	2.2	0.248	105 (15)	−0.4	0.858	97 (14)	−0.9	0.734
Independence	89 (14)	4.4	**0.010**	92 (15)	3.8	**0.044**	88 (16)	0.7	0.774	92 (15)	−3.1	0.224
INTERPERSONAL	107 (10)	1.8	0.255	106 (12)	0.3	0.863	106 (10)	2.1	0.330	102 (13)	−1.5	0.536
Interpersonal relationships	105 (12)	−0.04	0.979	104 (12)	2.0	0.279	104 (12)	0.3	0.914	102 (14)	−1.1	0.666
Empathy	107 (11)	2.8	0.162	106 (12)	−0.9	0.634	109 (10)	1.8	0.441	102 (13)	−1.9	0.468
Social responsibility	106 (11)	2.2	0.191	105 (12)	−0.1	0.954	101 (10)	4.1	**0.079**	100 (12)	−0.4	0.875
DECISION MAKING	97 (14)	3.6	**0.023**	100 (14)	3.6	**0.039**	99 (12)	2.4	0.258	98 (14)	−0.4	0.881
Problem solving	90 (14)	2.5	0.136	92 (16)	4.9	**0.009**	88 (15)	2.8	0.223	91 (15)	1.2	0.652
Reality testing	99 (13)	5.2	**0.002**	98 (14)	1.7	0.378	98 (13)	2.1	0.370	97 (15)	−2.7	0.289
Impulse control	105 (14)	1.1	0.505	102 (16)	1.7	0.371	103 (14)	0.8	0.720	94 (16)	0.8	0.754
STRESS MANAGEMENT	96 (13)	2.2	0.163	97 (16)	2.4	0.158	92 (13)	1.8	0.391	95 (15)	−1.1	0.638
Flexibility	96 (13)	2.3	0.178	97 (16)	1.5	0.420	94 (11)	2.5	0.275	96 (15)	−0.8	0.769
Stress tolerance	92 (14)	0.8	0.655	92 (17)	2.8	0.130	87 (14)	1.2	0.616	94 (15)	−0.6	0.798
Optimism	102 (13)	2.3	0.178	102 (14)	1.6	0.379	99 (13)	1.1	0.628	99 (14)	−1.9	0.456
Happiness	104 (12)	2.1	0.214	102 (15)	1.4	0.444	101 (13)	−0.5	0.844	100 (15)	−0.8	0.754

^X^p-value represents the significance of the change in EI score from T1 to T3. Changes in scores are calculated as T3 minus T1

Bolded p-values indicate a significant positive change occurred from T1 to T3

At T1, the Shapiro-Wilk test showed that 18 of the 22 scales were significantly non-Normally distributed, therefore comparisons between student groups at baseline was performed with non-parametric tests. Within the three therapy programs, there was only a significant difference in social responsibility (Kruskal-Wallis test Chi-square = 6.86, F = 2; *p* = 0.03), where the occupational therapy students appeared to score higher than the speech pathology students while all other scales showed no significant differences (all *p*-values> 0.2). The p-values shown in Table 2 were obtained from the ANOVA (total EI score) or random effects regression models (Subscale and Composite scores). As they were similar with respect to most scores, occupational therapy, speech, and physiotherapy students were combined into a single ‘therapy’ group for the purpose of comparison with business students.

By T3, mean EI scores for each student cohort were within the normal range (between 90 and 110). Occupational therapy students reported seven EI skills that improved significantly over the 16-month period: Total EI score, self-perception, self-actualisation, emotional self-awareness, independence, decision-making, and reality testing. Physiotherapy students showed significant improvement in self-actualisation, independence, decision-making and problem-solving. Speech pathology showed no significant changes in any EI skills. Business students reported no significant changes in any of the EI Subscale and Composite scores.

Table 3 presents the results of the ANOVA and two random effects regression models showing changes in the EI of all therapy and business students over the 16-month period, as well as a comparison of both groups. All therapy students showed significant increases in Total EI from T1 to T3, as well as the Composites of self-perception, self-expression, decision-making, and stress management. The therapy students Subscale scores showed significant increases in self-regard, self-actualization, independence, problem-solving, reality testing, and flexibility, with no EI scores declining.

**Table 3:** changes in the EI from T1 to T3 within an between all therapy and business

	Mean change		p-value ^X^ (between therapy and business)	p-value ^X^ (change from T1)	
	All therapy (*n* = 126)	Business (*n* = 20)		All therapy	Business
**Model 1**: Change from T1 to T3 in the Total EI score					
Total EI Score	2.7	−1.7	**0.015**	**0.005**	0.441
**Model 2**: Change from T1 to T3 in EI composite scores					
Self-perception	2.8	−1.3	0.104	**0.005**	0.576
Self-expression	2.0	−2.4	0.085	**0.046**	0.312
Interpersonal	1.3	−1.5	0.273	0.188	0.533
Decision making	3.3	−0.4	0.150	**0.001**	0.880
Stress management	2.2	−1.1	0.195	**0.031**	0.636
**Model 3**: Change from T1 to T3 in EI subscales scores					
Self-regard	2.2	−1.4	0.195	**0.047**	0.582
Self-actualisation	3.5	−0.2	0.183	**0.002**	0.937
Emotional self-awareness	1.2	−1.6	0.318	0.270	0.542
Emotional expression	0.9	−1.4	0.410	0.397	0.595
Assertiveness	0.0	−0.9	0.756	0.993	0.738
Independence	3.3	−3.1	**0.021**	**0.003**	0.222
Interpersonal relationships	0.7	−1.1	0.505	0.499	0.665
Empathy	1.1	−1.9	0.286	0.317	0.466
Social responsibility	1.8	−0.4	0.421	0.097	0.875
Problem solving	3.4	1.2	0.410	**0.002**	0.651
Reality testing	3.3	−2.7	**0.032**	**0.003**	0.288
Impulse control	1.3	0.8	0.870	0.256	0.753
Flexibility	2.1	−0.8	0.308	**0.060**	0.768
Stress tolerance	1.6	−0.7	0.422	0.153	0.798
Optimism	1.8	−1.9	0.181	0.103	0.455
Happiness	1.3	−0.8	0.450	0.242	0.753

Bolded p-values indicate a significant change from T1 to T3

**Fig. 2:**
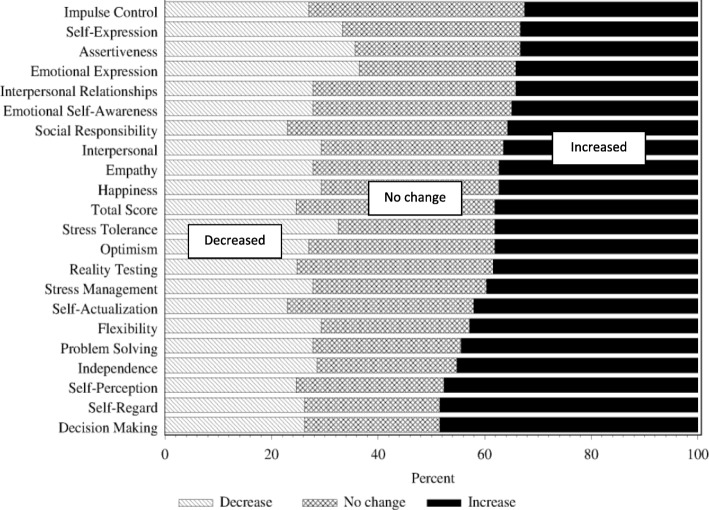
percentage of all therapy students who decreased, remained the same or increased their EI score from T1 to T3

Figure 2 shows the percentage of all therapy students whose EI scores increased or decreased (by five points or more) or remained the same over the 16-month period. The EI skills where a large percentage of students increased their scores were decision-making (48%), self-regard (48%), self-perception (47%) and independence (44%). Scores decreased in a large percentage of therapy students in emotional-expression (38%), assertiveness (37%), stress tolerance (32%), and self-expression (32%).

## Discussion

The study contributes to the understanding of the changing EI skills of therapy students. The findings support our hypothesis that therapy students’ Total EI would increase significantly over the period that coincided with full-time, extended clinical placements. Our study adds considerable depth to understanding the strengths and shortcomings of therapy students’ EI skills as they complete their full-time, extended placements. The findings that *Total EI*, as well as most EI skills improved while students participated in clinical settings, should be reassuring to clinical educators and university educators, and validate the critical role that clinical placements have in the transition of the student therapists to practising therapist. At the same time, nine composite and subscale EI scores increased significantly amongst therapy students. The hypothesis that business students, who do no placements, would show no changes in EI scores, was supported. We cannot state that improved EI competencies are the direct result of clinical placements, as part of the change may have been due to natural emotional maturation that occurs over time, or personal life events external to the placements [30]. However, given that Total EI and some subscale EI scores increased in therapy students, while business students showed no improvement, it is reasonable to infer that clinical placements are a key influence on some EI competencies.

The increase in Total EI and subscale scores demonstrate that an array of competencies central to being an effective therapist improve over the period that full-time clinical placements occur. Our study’s findings parallel those of Clarke [18] who reported that EI competencies could be enhanced during workplace learning. Clarke reported that emotional awareness and management influenced students’ critical reflection as well as social engagement and conflict management. Therapy students’ EI competencies may have improved because of the daily interactions with patients in vulnerable situations where students had to manage their own and the patient’s emotions. Clinical placements immerse the student in a range of emotion-based scenarios on a daily basis where students must show competence in these scenarios. Placements also offer the opportunity for clinical supervisors to give students feedback and encourage the student to reflect on their EI abilities. Thus, students must adapt and enhance their EI skills during placements, otherwise they might be at risk of failing the placement.

Our study’s findings are contrary to the Lewis’ [22] 3 year study of physiotherapy students who found no significant change in Total EI or subscales but did report that EI was minimally correlated to performance during placements. However, Lewis’s study used an ability-based measure. Larin et al.’s [23] study of nursing and physiotherapy students used a mixed-model measure (Emotional Quotient Inventory: Short) and measured EI from before to after their first clinical placements, a period of 1 year. This study found no significant change in EI scores. Emotional intelligence in our study was measured at three distinct points over a 16-month period during which students were primarily undertaking full-time placements.

Of concern were the large proportion of therapy students whose emotional-expression, assertiveness, self-expression, and stress tolerance scores decreased over the 16-month period, attributes that are critical to being an effective therapist and member of a healthcare team. Prior to their full-time placements, some students may feel confident of being assertive and able to manage their stress. However, they soon realize that the healthcare workplace is a difficult setting to be assertive for novice practitioners, and there is an array of daily stressors to deal with, compared to life as a university student. Clinical placements might have a negative influence on EI abilities if the student experiences poor quality supervision. Grenier [35] reported that clinical supervisors who demonstrate poor communication skills, disengagement, high levels of control, being closed-minded or supervised with intimidation could negatively impact a student’s performance. Gribble et al. [27] also reported that clinical supervisors could have a negative impact on the EI competencies of therapy students.

### Implications for clinical supervisors, university educators and employers

The findings that Total EI, as well as some Subscales, improve while students are immersed in healthcare settings, should reassure clinical supervisors and university educators. Increased EI scores may result in a therapy student being able to show enhanced empathic behaviours, deal with complex emotional scenarios independently, perform well in team interactions, and ultimately making better clinical decisions.

Clinical supervisors should be cognizant that the EI of some students may be lower than other students, especially during the student’s initial full-time placements. Supervisors should take this into account when evaluating a student’s performance. Supervisors and employers should also be aware that a student’s EI competencies may not be fully developed by the completion of the university program, but should continue to mature as they enter the workforce. Workshops on EI skills, peer coaching, and mentoring programs may be useful to support the development of EI in recent graduates [36, 37]. Further longitudinal research could track new graduate therapists through their first few years of practice to identify if, and when, the EI constructs change during this transition to benchmarked levels of EI for practising therapists.

Low or decreasing EI scores may impact a student’s performance and may result in the student being graded as failing the placement. For example, a student with low assertiveness and self-expression might be passive in team meetings and lack decisiveness when they communicate with patients. Students low in independence may be passive during emotional scenarios and turn to their supervisor or colleagues for strategies to deal with the scenario [38]. Students who experience difficulty during placements have been reported to demand additional time from supervisors [39], thus students with lower EI scores may also require additional support, although more research is required ot confirm this possibility. Supervisors need to be aware that a poor supervisory environment might impact a student’s EI skills [27]. For example, an intimidating or disengaged supervisor might diminish a student’s assertiveness and self-expression, which consequently impacts the student’s performance.

Various authors [19, 20] have suggested that training of EI competencies be scaffolded throughout the healthcare curricula. Stoller et al. [40] suggest that EI should not be taught as a stand-alone module, but integrated and revisited with increasing sophistication throughout the curriculum. Integration of EI into healthcare curriculum may result in enhanced EI abilities of students as they commence their first full-time placements, and more importantly when they commence work as new graduates.

### Limitations

A control group of therapy students would have been preferred to business students but was not possible given the obligatory requirement to undertake clinical placements. Attrition of participants between the various phases was a limitation of the study, and especially in the business students there was a lack of power and the possibility of a Type II error. In particular, the number of business students who responded at T3 was small and could lead to potential bias in their mean EI change. Because the EQ-i^2.0^ is a self-report instrument that measures the participant’s perception of their EI abilities, this study cannot purport that EI skills have improved, only that student’s perceptions of their EI competencies have improved. Future studies could use the EQ-i:360 where peers and supervisors actually evaluate the observed EI competencies of the students.

## Conclusions

Emotional intelligence is a critical skill for occupational therapists, physiotherapists, and speech pathologists that should be actively fostered during clinical placements and when they first enter the workforce through mentoring, peer coaching and training. Total EI, as well as some EI skills, improve while students participate in healthcare settings because of the daily interactions with patients in distress, pain or vulnerable situations. Clinical supervisors should be aware that the EI of some students may be lower than other students, especially during the student’s initial full-time placements and supervisors are encouraged to take this into account when they evaluate a student’s performance. Clinical supervisor training should highlight the substantial positive and negative impact that supervisory style can have on the EI competencies of therapy students. Clinical supervisors are encouraged to give students ongoing feedback about their EI abilities, as well as their practical and clinical reasoning skills. Equipping therapy students with more mature EI skills may ultimately result in stronger clinical placement performance and superior graduates as they enter the workforce.

## Abbreviations

EI: Emotional intelligence
EQ-i^2.0^: Emotional Quotient Inventory version 2.0
